# Unlocking stray light mysteries in the CoRot baffle with the time-of-flight method

**DOI:** 10.1038/s41598-024-56310-z

**Published:** 2024-03-14

**Authors:** L. Clermont, P. Blain, W. Khaddour, W. Uhring

**Affiliations:** 1 Centre Spatial de Liège, STAR Institute, Université de Liège, Avenue du Pré-Aily, 4031 Liège, Belgium; 2 ICube Research Institute, University of Strasbourg, 23 Rue du Loess, 67037 Strasbourg Cedex, France

**Keywords:** Applied optics, Optical techniques, Techniques and instrumentation

## Abstract

Stray light (SL) has emerged as a primary limiting factor for space telescopes. Pre-launch testing is essential for validating performance and identifying potential issues. However, traditional methods do not enable the decomposition and identification of individual SL contributors. Consequently, when problems arise, resolving them often involves a cumbersome and risky trial-and-error approach. The time-of-flight (ToF) method was recently introduced, employing a pulsed laser source and ultrafast sensor to characterize individual SL contributors. A proof of concept was achieved using a simple three-lens system. In this paper, we apply the ToF method to a real space optical system: the spare model of the CoRoT baffle. We successfully measured individual SL contributors over a dynamic range of 10^−11^, identifying direct scattering on vane edges and two-step scattering paths. Our results provide a performance breakdown, differentiating intrinsic baffle SL from contributions arising from experimental conditions. Notably, the ToF method allowed us to discriminate air scattering, eliminating the need for expensive vacuum testing. The ToF provides unparallel insights, including defects identification. For instance, we identified the presence of localized dust particles causing significant SL. These results confirm the utility of the ToF method even for the most challenging space systems.

## Introduction

In the quest to develop ever-more advanced space telescopes, stray light (SL) increasingly emerges as a primary limiting factor. SL, which arises from ghost reflections and scattering, degrades image quality and introduces artifacts^1,2^. This is especially problematic for telescopes tasked with observing faint objects or small variations in their intensity. A notable example is the CoRoT mission, with the objectives of conducting asteroseismology measurements and searching for exoplanets. Launched in 2006, its unique 30 cm diameter telescope has successfully observed variations of star intensities as subtle as one part in one hundred thousand^3,4^.

An external baffle is employed to prevent SL from entering the telescope^1^. The CoRoT baffle stands out as one of the top-performing configurations^5–7^ and has served as an inspiration for missions such as CHEOPS^8^. Vanes are strategically arranged inside two successive cylinders, forcing light to experience multiple scattering events before it can reach the telescope's entrance^2^. Coated with a black treatment and featuring razor-thin edges, the baffle reduces the SL by several orders of magnitude. Ray tracing simulations predicted a SL attenuation down to 10^−6^ at the baffle's exit plane for a single angle illumination, resulting in an impressive 10^−11^ attenuation at the telescope's focal plane^7^. Such extreme rejection is necessary as SL comes from combined illumination at all angles. Although the baffle was not experimentally tested on-ground, its in-space performance aligned with expectations. However, bypassing ground testing was a considerable risk, and today, such testing is deemed essential^9–12^. In fact, several space instruments, including GAIA^13^, TIRS^14,15^, and more recently Euclid^16–18^, have encountered unexpected SL issues after being deployed in space. When this happens, only limited mitigation methods can be implemented^13^, making SL a potential cause of mission failure.

On-ground testing aims to identify deviations from expected performance^1,19,20^. Should discrepancies arise, the design must be refined to meet the requirements before the payload is launched. However, traditional testing methods are limited as they only provide a binary outcome: pass or fail. When the system under test is illuminated, the measured SL contains the combined effect of SL contributors originating from various paths. With no way to decompose and retrieve the origin of individual contributors, identifying the root cause of a SL issue is not trivial. Refining the design can only be achieved through a cumbersome and uncertain trial-and-error process^1^. Moreover, the measurement can include SL caused by the testing facility itself, such as scattering by the air. As a result, SL measurements frequently need to be conducted under vacuum conditions^19,20^.

To address to these challenges, Clermont et al.^21^ introduced the time-of-flight (ToF) technique for SL characterization. Using a pulsed laser combined with an ultrafast sensor, this method discerns SL components through their unique optical paths, each manifesting as a distinct time-of-flight. Such differentiation not only allows for precise individual measurements but also enables the pinpointing of the origin of each contributor based on their arrival times at the detector. The ToF method was showcased in characterizing a small refractive telescope with a dynamic range of 10^−9^. Employing a femto-second laser paired with a streak camera, the system records photon arrival times along a unidimensional slit^22^, distinguishing optical path differences at the millimeter scale. A subsequent scan of this slit unveiled the intricate 2D spatial distribution of individual ghost components^21^.

In a subsequent experiment, the ToF method was employed to verify the performance of a conventional SL facility^23^. Because conventional testing facilities must surpass the precision of the instruments they evaluate, their verification necessitates an even superior measurement device^19,20^. The ToF technique distinguishes contributions from the measurement device based on their individual times-of-flight, enabling the extraction of the facility's intrinsic signature. This approach was pivotal for the Earth Return Orbiter (ERO) NAC test campaign and is slated for future applications such as the FLEX mission^11^. A pico-second laser, paired with a single-photon avalanche diode^24^ (SPAD) in combination with the time correlated single photon counting method (TCSPC), was utilized to resolve optical path differences as small as a few centimeters. With a single pixel SPAD, a 2D scan was conducted. In the future, the ToF will benefit from the recent improvement of SPAD arrays with increasing numbers of pixels^25,26^, potentially eliminating the need for scanning.

Interferometry offers an alternative method to discern SL paths with finer optical path length differences^27,28^. Light-in-flight holography, for example, allows the recording of pulse propagation through spatial multiplexing^30–36^. Another method is the frequency-modulated continuous-wave (FMCW) method^29^, which Takakura et al. utilized in the THz spectral range for the LiteBIRD mission^37,38^. In the context of the LISA mission, Khan et al.^39^ employed low-coherence interferometry to characterize back-scattering at optical interfaces. However, interferometry requires a more intricate setup including a reference beam, not adapted to complex optical systems^27^.

In this paper, we explore the application of the ToF method in characterizing the spare model of the CoRoT baffle—a benchmark in high-performance SL baffles. Theoretical studies have indicated that scattering within a baffle could be differentiated based on their optical path lengths with a ToF setup^27^. Our goal is to provide a detailed breakdown of the SL performance by each contributor and, where possible, pinpoint design flaws in the baffle. Given the baffle's large size—over a meter—optical path differences are expected to reach several tens of centimeters or even greater. In this context, the SPAD emerges as the most appropriate technology for the task^21,23,27^. As opposed to previous applications, the SL output from a baffle is uniform^27^, making a single-pixel SPAD, without the need for scanning, adequate. However, to accommodate the large entrance aperture of the baffle, a spatial scan of the input beam is necessary^9,27,40^.

## Results

### Experimental setup

**Figure 1:**
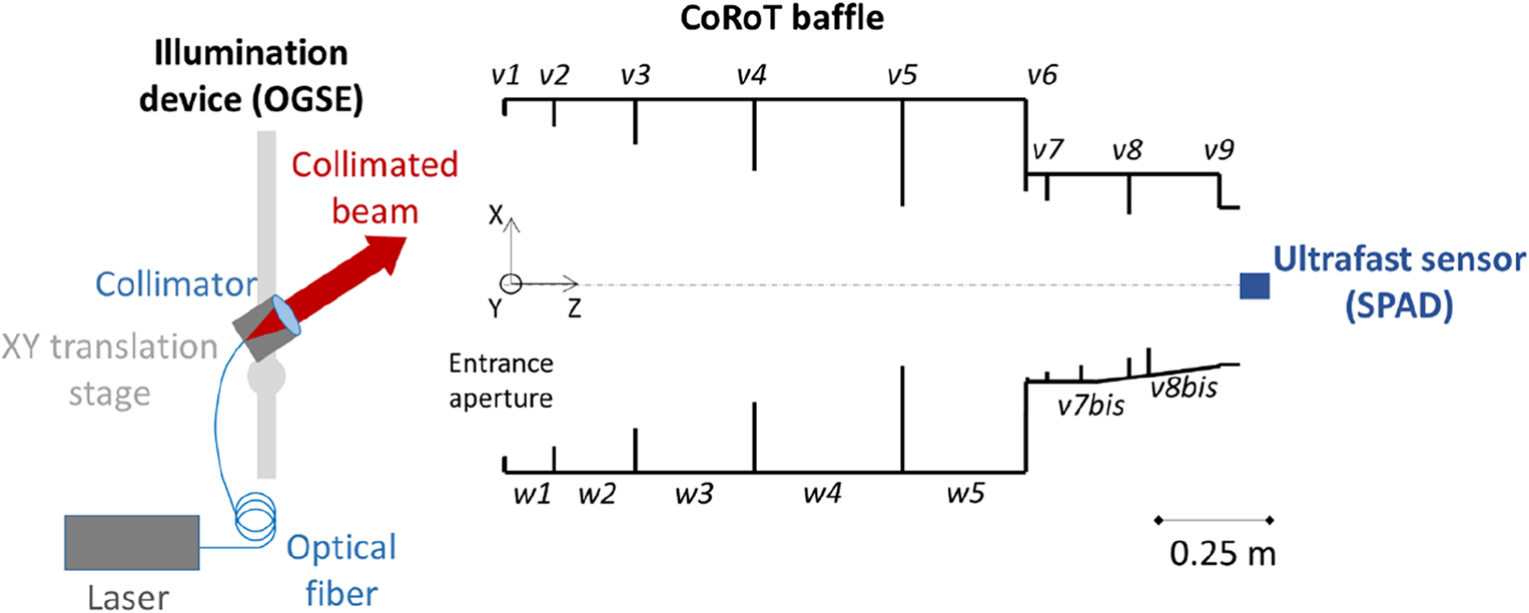
Sketch of the experimental setup.

The experimental setup is shown in Fig. 1. It comprises an illumination device known as the OGSE (Optical Ground Support Equipment), the CoRoT baffle under examination, and an ultrafast sensor. Inside the CoRoT baffle, the vanes are labeled 'vi' and the cylinder walls as 'wi'. The OGSE utilizes a 532 nm ps-pulsed laser connected to an optical fiber. The fiber’s tip is placed at the focal point of a collimator, producing a 25 mm collimated beam at a field angle of 33°. Mounted on an XY translation stage, the collimator scans the baffle's input aperture, encompassing SL from diverse paths as various elements are illuminated^27,40^. The SPAD ultrafast sensor is placed at the exit plane of the baffle to capture the SL. For each scan position, a measurement is performed with the SPAD. For each measurement, the signal *S*(*t*) is normalized to the direct light from the collimator.

### SL movie

**Figure 2:**
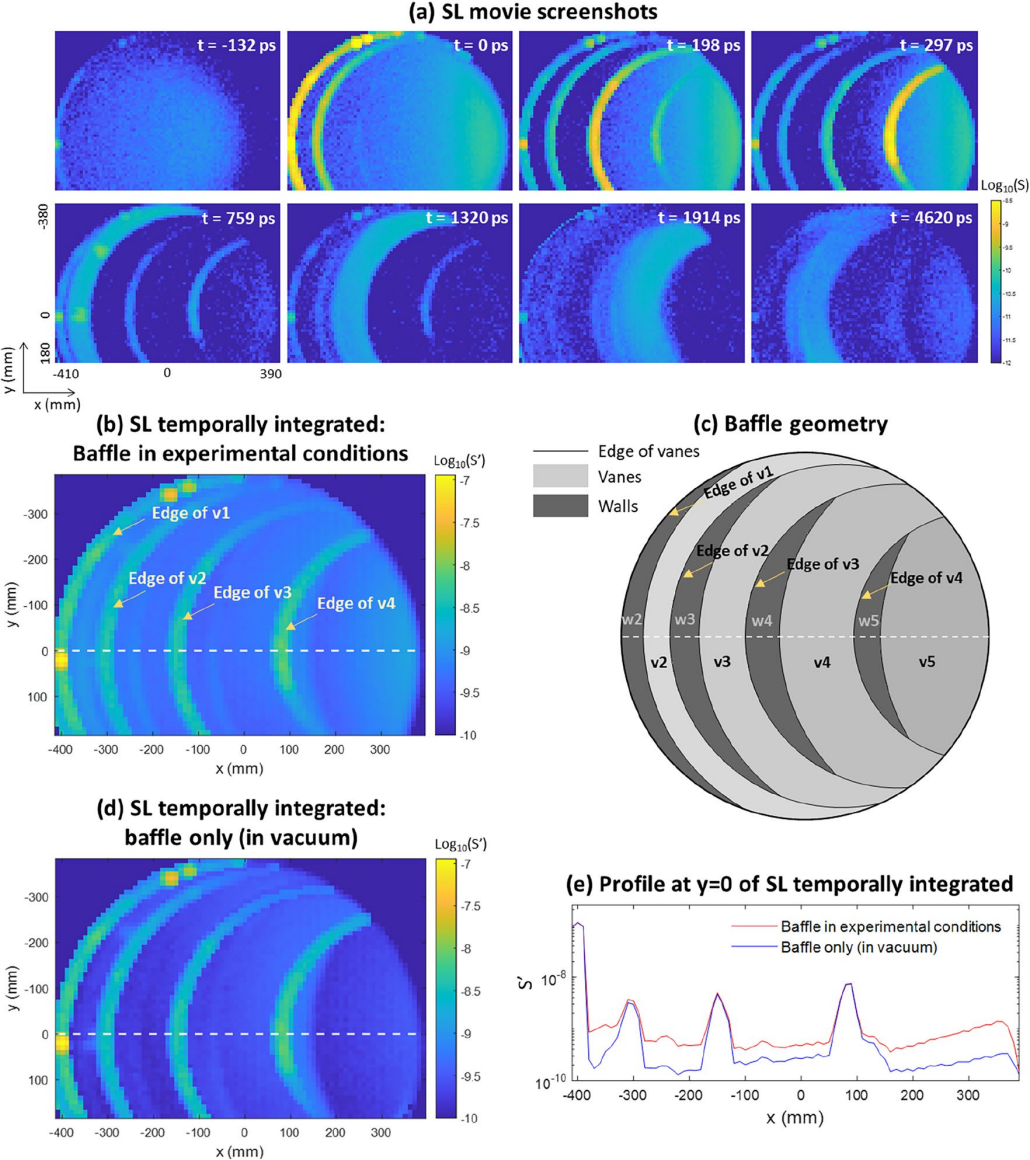
(**a**) Screenshots of the SL movie. (**b**) Temporal integration of the SL movie. (**c**) Baffle geometry. (**d**) Temporal integration of the SL movie, excluding the contributions from OGSE SL and air scattering. (**e**) Profiles along y = 0 of the SL movie temporally integrated.

By assembling the measurements from various scan positions (*x*,*y*), we obtain a chronological sequence of 2D maps, represented as *S*(*x*,*y*,*t*). This sequence creates a temporal movie of the SL reaching the SPAD as a function of the illuminated position on the baffle. The complete movie can be found in the supplementary materials. Figure 2a shows selected screenshots captured at different times. Figure 2b showcases the movie temporal integration, $S^{\prime } \left( {x,y} \right) = \sum\nolimits_{t} S \left( {x,y,t} \right)$. Due to the limited range of the translation stage, the bottom part of the baffle aperture is missing.

Figure 2c illustrates the baffle's projected geometric layout, indicating which element is illuminated for a given (*x*,*y*). Notably, direct SL from vane edges is observed on the screenshots for times t = 0 ps 198 ps and 297 ps. At both t = 1320 ps and t = 4620 ps, SL detection associated with v3 illumination results from a two-step scattering process: light initially interacts with v3 and subsequently with another surface before arriving at the sensor. The difference in time suggests that the subsequent surfaces for these two paths are at different distances.

### SL cross-section

**Figure 3:**
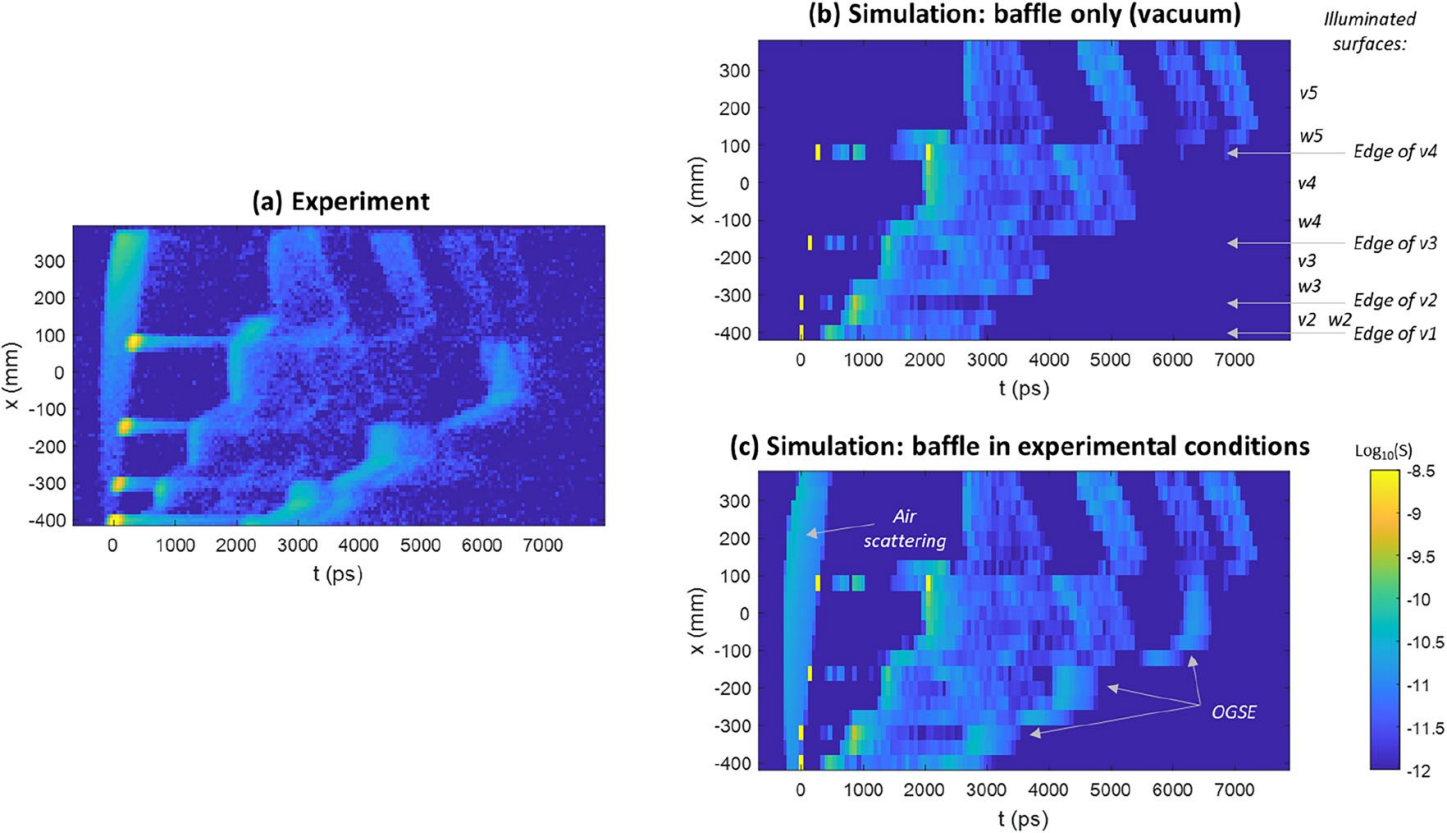
Cross-section of the SL movie along *x*, for *y* = 0. (**a**) Experimental result. (**b**) Simulation result with baffle only. Arrows indicate the illuminated element for a given *x* value. (**c**) Simulation in experimental conditions, including air and OGSE scattering.

Figure 3a shows a cross-section of the movie along *x*, for *y* = 0. In comparison, Fig. 3b depicts the outcome from a ray tracing simulation for the baffle in vacuum. The experimental results reveal several features absent in the simulation. When incorporating scattering properties of both the OGSE and air, effectively replicating the baffle's experimental conditions, the simulation result shown in Fig. 3c aligns well with the experimental observations.

### Baffle intrinsic SL

This analysis provides clarity on which portions of the measurement arise from the experimental conditions versus those inherent to the baffle. For instance, the background scattering observed in the screenshots from t = − 132 ps to 297 ps in Fig. 2a is due to air scattering. Also, the SL path at t = 4620 ps upon illuminating v3 results from a secondary scattering effect on the OGSE. Consequently, we can perform temporal integration of the movie while excluding contributions from OGSE and air scattering, as demonstrated in Fig. 2d. A profile is also shown in Fig. 2e.

### Instrument SL performance breakdown

By spatially and temporally integrating the SL movie, we determine the total SL captured by the sensor, corresponding to the definition of the Point Source Transmittance (PST). The PST is a single number whose value is compared to the performance requirements. By selectively integrating the SL movie over specific spatial coordinates and time intervals, we can effectively decompose the PST based on its various contributors. This is presented in Table 1, with the left columns showcasing the PST breakdown from the measured aperture. The right column provides results for the full aperture, estimating the missing segment of the aperture by symmetry. A simple mirror symmetry is performed on the SL movie, attributing to the missing scan values (Y > 0) the corresponding signals from the other side (Y < 0). Then, the spatial and temporal integration is performed the same way as for the original movie.

**Table 1:** Point source transmittance breakdown.

Contributor	Point source transmittance (PST)			
	Measured aperture		Full aperture	
	Absolute	Relative (%)	Absolute	Relative (%)
**Air scattering**	**6.0E−07**	**11.2**	**7.2E−07**	**10.6**
**OGSE scattering**	**7.6E−07**	**14.0**	**9.7E−07**	**14.3**
**Baffle intrinsic SL:**				
Scattering on edge of v1	1.8E−06	34.1	2.1E−06	31.8
Scattering on edge of v2	3.6E−07	6.6	4.9E−07	7.3
Scattering on edge of v3	4.0E−07	7.4	4.5E−07	6.6
Scattering on edge of v4	4.8E−07	8.8	6.7E−07	9.9
Total	**3.1E−06**	**56.9**	**3.7E−06**	**55.5**
**Baffle intrinsic SL**:				
Scattering on v2/w2 then on v1	1.1E−07	2.1	1.7E−07	2.5
Scattering on v3/w3 then on vi (i = 1:2)	2.0E−07	3.7	3.0E−07	4.4
Scattering on v4/w4 then on vi (i = 1:3)	3.5E−07	6.5	5.0E−07	7.4
Scattering on v5/w5 then on vi (i = 1:4)	3.0E−07	5.6	3.5E−07	5.2
**Total**	**9.7E−07**	**17.9**	**1.3E−06**	**19.5**
**Total: baffle in experimental conditions**	**5.4E−06**	**100.0**	**6.7E−06**	**100.0**
**Total: baffle intrinsic SL**	**4.0E−06**	**74.8**	**5.1E−06**	**75.0**

The breakdown differentiates the effect of air and OGSE scattering from the baffle's intrinsic SL. The latter is further decomposed into several contributors, including the effect of direct scattering on the edges of individual vanes. Subsequently, the table breaks down the SL paths associated with the illumination of various pairs vi/wi. Upon illumination of v2 or w2, light undergoes an initial scattering, followed by a secondary scattering on v1, before ultimately reaching the SPAD. Illuminating a vane or wall deeper within the baffle results in multiple SL paths. For instance, when illuminating v5 or w5, four distinct paths arise due to a second scattering on one of the subsequent vanes v1 to v4. With distinct times-of-flight, as depicted in Fig. 3, these could have been decomposed. In contrast, multiple paths originating from a first scattering on v4/w4 or v3/w3 cannot be decomposed due to overlapping times-of-flight.

Table 1 indicates that a substantial 25% of the total measured SL originates from the experimental conditions. Notably, the intrinsic baffle’s PST is 5.1 × 10^−6^, a value very similar to the simulation prediction^7^ of 3 × 10^−6^. Furthermore, the table highlights the significant impact of scattering on the edges, with a particularly pronounced contribution from the edge of v1.

**Figure 4:**
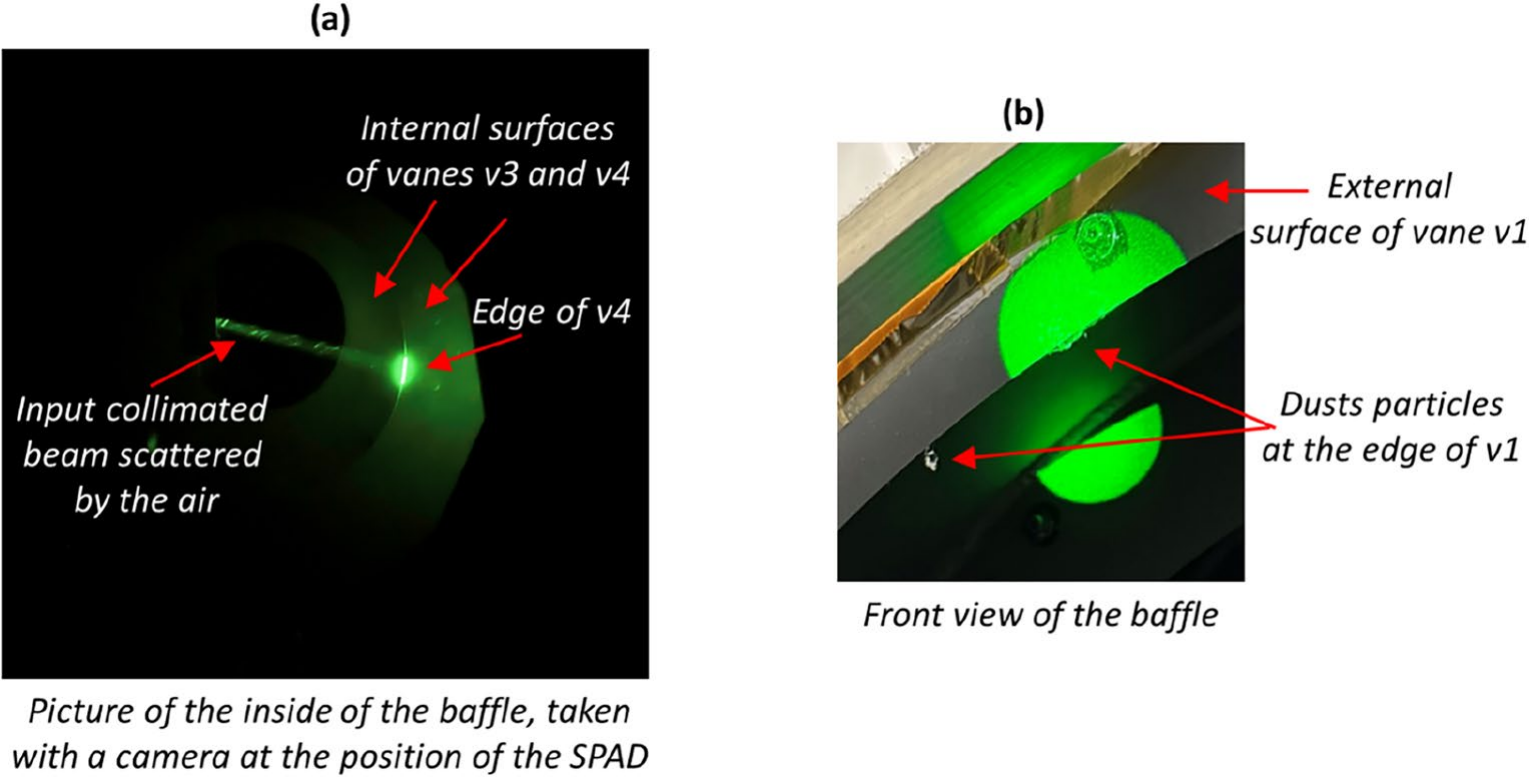
(**a**) Image taken with a conventional camera, positioned at the typical SPAD location, highlighting air scattering, direct edge scattering, and two-stage processes on the vanes. (**b**) Detection of dust particles on the edge of vane v1.

Figure 4a presents an image captured using a conventional camera at the typical location of the SPAD. It showcases various SL contributors within the baffle, with the input beam illuminating the edge of v4. While air scattering is visible, the OGSE, being obscured in this scan position, does not generate SL. Most prominently, the bright direct scattering on the edge of v4 stands out. Additionally, we detect SL emanating from the bottoms of v3 and v4, resulting in two-step scattering processes with a first interaction on the top of v4 or v5. Finally, scattering on the edge of v4 also produces SL through a second scattering process on subsequent edges. While it cannot be observed in the image due to its faintness, this effect is visible in the SL movie or in the cross-section of Fig. 3a, where the bright direct SL is followed by a fainter tail.

### Identification of design defects

The significance of SL from the edge of v1 in Table 1 prompted a closer examination. Upon inspecting the SL screenshot at t = 0 ps and the movie's temporal integration (see Fig. 2), three distinct bright spots are apparent along that edge. Their simultaneous appearance alongside scattering from other parts of that edge suggests they are direct SL effects, stemming from localized defects on v1. Knowing the (x,y) position of these defects, we examined the vane v1, as depicted in Fig. 4b and two small dust particles were found its edge. Despite their small size, these particles have a significant influence on the SL. Removing them reduces the SL from the edge of v1 from 34.1 to 6%, comparable to the levels seen on the other edges. While working in a cleanroom generally prevents the presence of dust, other common defects, such as scratches, could similarly be identified.

### Baffle hemi-reflectance estimation

**Figure 5:**
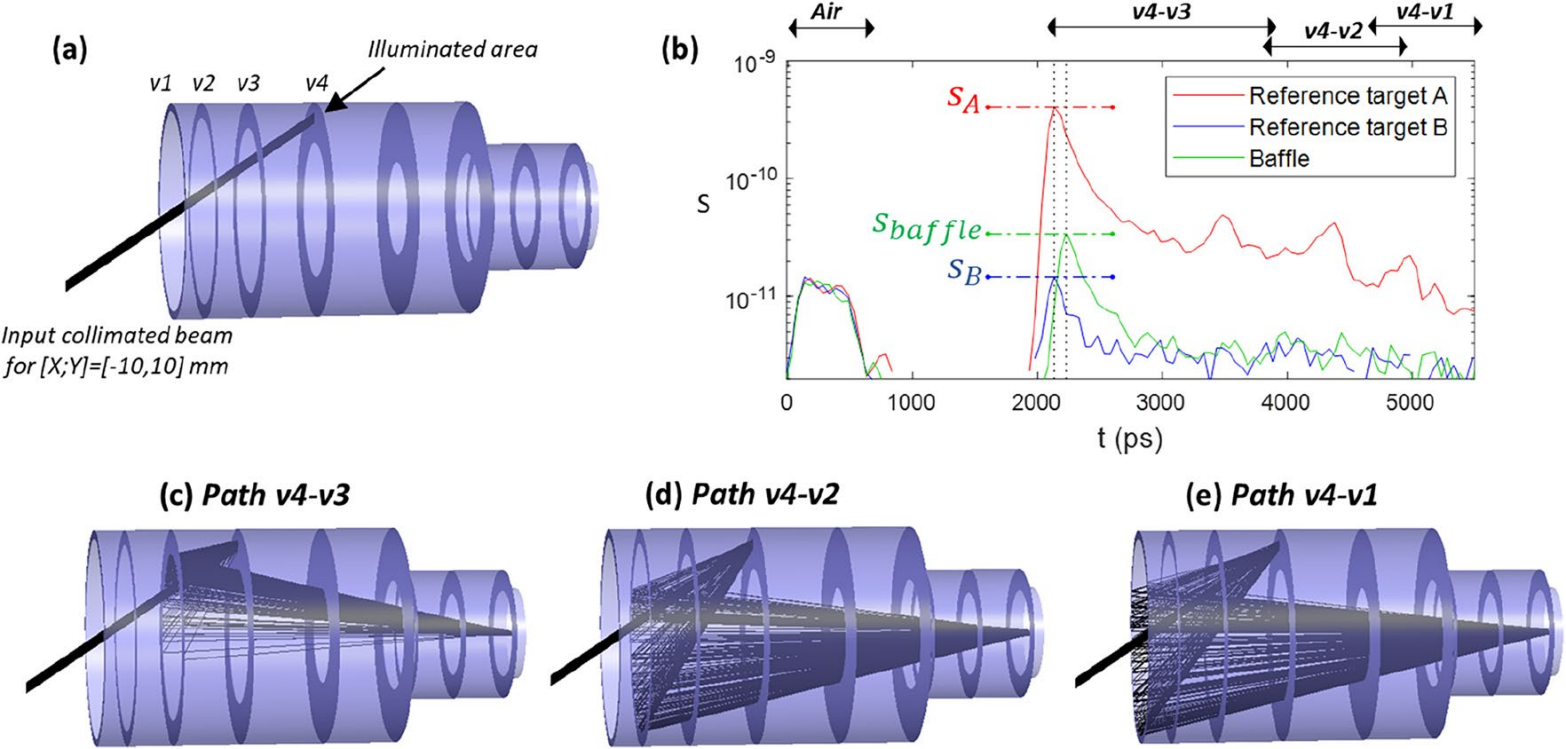
(**a**) Ray tracing depiction of v4 illumination. (**b**) SL measurement upon vane v4 illumination, with and without a reference target. The ratio s_baffle_/s_A_ or s_baffle_/s_B_ enables the deduction of R_baffle_ (**c**–**e**) Ray tracing illustrations of the different SL paths.

The ToF technique can be employed to estimate the hemi-reflectance of the baffle’s black treatment, represented as R_baffle_. As depicted in Fig. 5a, the area to probe is illuminated, and a spectrum *S*(*t*) is captured. The measurement is then repeated in the same area using a reference target with known hemi-reflectance values of R_0_ = 90% or 2%. The results shown in Fig. 5b encompass SL paths that start with an interaction on v4 and continue with a second scattering effect on the preceding vanes, as detailed in Fig. 5c–e. The signal from these SL paths is directly proportional to the hemi-reflectance of the probed area, allowing the deduction of R_baffle_ by comparison with the reference. Consistent estimations of 4.6% and 4.2% are obtained with the 90% and 2% reference targets, respectively. These estimations align with expectations for CoRoT.

It is noteworthy that a − 100 ps shift in the SL profile is observed for the reference measurements, which corresponds to an optical path length reduction of approximately 30 mm. This shift can be attributed to the target's thickness of 13.97 mm.

## Discussion

In this study, for the first time, we utilized the ToF method to characterize a space optical system: the CoRoT baffle. Using selective temporal and spatial integrations of the SL movie, we derived a breakdown of the SL contributors. This approach enables us to differentiate between the intrinsic SL of the baffle and SL resulting from experimental conditions. Notably, scattering from the OGSE and air accounts for 25% of the total SL. Scattering on the edges of the vanes is the primary source of SL within the baffle, followed by two-step scattering processes between its various components. Dust particles were identified on the edge of v1, suggesting that other defaults or imperfections such as scratches could be pinpointed in an instrument. Additionally, we verified the hemi-reflectance of the baffle's black treatment.

The capability to break down SL performance offers unparalleled insights into a system's SL properties. It not only highlights the most significant contributors but also facilitates the identification of potential defects. Consequently, engineers can address these defects with targeted solutions, bypassing the pitfalls of a trial-and-error approach. Sometimes, an underperforming system doesn't stem from one major issue but rather from the combination of smaller problems that are easier to address when pinpointed. Furthermore, distinguishing SL resulting from experimental conditions eliminates uncertainties over potential measurement artifacts. This enables the use of a measurement device with a residual SL that might be worse than the instrument under test, while still ensuring confidence that the detected SL originates from the instrument itself. Finally, the capability to suppress the air scattering avoids the need for expensive and unpractical vacuum testing.

Spatially scanning the entrance aperture offers a practical alternative to constructing a massive collimator to illuminate the full instrument at once. Moreover, combining spatial and temporal decompositions enhances the differentiation of paths and assists in identifying potential design flaws. However, this method extends the duration of the test campaign. In the future, the ability to adjust the beam size, for instance using a simple iris aperture, would be advantageous. An initial scan could be conducted with the widest beam setting, followed by more detailed scans in areas requiring higher spatial resolution, using a narrower beam and smaller sampling steps.

SL properties in optical systems are a function of the angle of incidence, with issues potentially manifesting at specific angles. Hence, SL is traditionally measured over different angles. Likewise, ToF characterization should be conducted at multiple angles to understand how the different SL contributors vary. Furthermore, SL properties exhibit spectral dependence, and an instrument's performance may vary across the spectrum. For example, a black treatment might effectively absorb visible light but falter in the Near-Infrared region, or vice versa. Although ToF measurements necessarily employ monochromatic light, ultrafast sensors with tunable lasers boast capabilities spanning a wide spectral range, extending from the visible to several microns in the IR. For instruments with a wide operational spectrum, ToF characterization should be executed at multiple wavelengths, ideally at both the spectrum's extremes and its midpoint. This underscores the imperative for automation, both in conducting the measurements and subsequent data analysis. Alternatively, spectral dependance could be evaluated more rapidly by measuring the hemi-reflectance of the baffle at another wavelength, and scaling the SL from the various paths accordingly.

The ToF method was first introduced as a proof of concept on a small three-lens system. In this study, we demonstrated its applicability to a larger, real-world system, specifically the CoRoT baffle, known for being one of the most effective SL baffles. Previously, the characterization focused on ghosts reflections between lenses. Here, we identified extremely faint SL features, originating from scattering effects inside the CoRoT baffle, with intensities as low as 10^−11^. Looking ahead, a logical progression would be to examine complete systems that incorporate both the baffle and the telescope. This would allow for a comprehensive characterization of SL complexities in an integrated system, encompassing SL from the telescope (including ghosts), scattering from the baffle, and SL produced by interactions between the baffle and the telescope.

Our findings underscore the ToF’s prowess in characterizing even the most challenging space systems. It offers enhanced accuracy, efficiency, and invaluable insights for design refinement. This will contribute to the development of advanced space telescopes with record-low SL levels, paving the way for new discoveries and scientific breakthroughs.

## Materials and methods

### Optical system under test

The optical system under test is the spare model of the CoRoT baffle. Its design is the same as the flight model which was sent in space. The baffle has a maximum diameter of 832 mm and an entrance aperture diameter of 770.6 mm. Its full length is 1652.9 mm. The baffle blocks the out-of-field light from entering the CoRoT telescope, placed at the exit of the baffle.

### Laser source

The laser is an OEM model (original equipment manufacturer) manufactured by the company *Multitel*. It is a 532 nm pulsed laser with repetition rate 10.48 MHz. The laser has an average power of 70 mW and a pulse duration of 3 ps.

### Ultrafast sensor

The ultrafast sensor is a single-photon avalanche diode (SPAD) with single-pixel of 50 µm size. The SPAD is paired with the laser with the Time-Correlated Single Photon Counting (TCSPC) system. An FPGA based Time digital converted (TDC) is used to measure the arrival time of the photon on the detector. The signal is sampled with a sampling of 50 ps, corresponding to the propagation of light in vacuum over 15.15 mm. The system impulse response has a full width at half maximum (FWHM) of 124.35 ps. This is the time for light to propagate over a distance of 37.68 mm and represents the smallest optical path length difference distinguishable.

### Illumination device

The illumination device consists of an aspheric lens collimator with focal length 101.6 mm and diameter 50.8 mm. The laser is injected at the collimator focal plane through an optical fiber of 50 µm core diameter. The input beam thus has a divergence of ± 0.014°. The collimator is placed on a translation stage with the capability to scan the system over 800 mm along X and 560 mm along Y. An angle of illumination of 33.1404° with respect to the baffle is selected. The choice of 33.1404° is because it is an out-of-field angle for the system, where critical light sources are typically located (such as the Earth or the sun). The pulse duration is slightly increased when passing through the optical fiber, this is negligible compared to the SPAD resolution.

### Acquisition and processing

Scan of the collimator is performed with steps of 10 mm. At each position, an acquisition is performed with the SPAD. For each acquisition, we average the temporal spectrum recorded at a frequency 10.41 MHz during 30 s. The measurements are normalized to the nominal. The nominal is obtained by illuminating directly the SPAD with the input beam, using an OD 8 optical density to avoid saturation. We set the arrival of the SL from vane 1’s edge as t = 0 (first SL effect produced by the baffle).

### Photon efficiency

In these experiments, we were not limited by photon-efficiency and we were able to measure SL features down to 10^−11^. For such a signal at 10^−11^, the SPAD receives 1 photon/s. Brighter features at 10^−7^ thus corresponds to 10.000 photon/s. An even better dynamic range could be obtained if necessary, by increasing the laser power. Furthermore, by increasing the integration time, we improve the signal to noise ratio and therefore also the dynamic range.

### Ray tracing simulations

The ray tracing simulations are performed with the software FRED, introducing the geometry of the CoRoT baffle. The elements in the baffle are assumed to scatter the light in a Lambertian way and with hemi-reflectance of 4%. Every ray is scattered up to two times before reaching the sensor. Higher orders rays have negligible contribution. When introducing the experimental conditions in the simulation, the mechanics of the illumination device is defined with the same scattering properties as the baffle. Air scattering is simulated with a simplified isotropic assumption and hemi-reflectance scaled on the experimental results.

### Baffle hemi-reflectance estimation

Baffle hemi-reflectance estimation is done with input beam on v3, with (x,y) = (− 10 mm, 10 mm). An acquisition is performed and compared with a reference obtained by placing at the illuminated area a target. The target is a 1 inch diameter and 1.5 mm thick Spectralon sample. It has an hemi-reflectance $R_{reference}$ of 90% or 2%. If the maximum SL signal is $s_{baffle}$ for the baffle, $s_{reference}$ for the target, then the baffle hemi-reflectance if obtained with $= R_{reference} \cdot \frac{{s_{baffle} }}{{s_{reference} }}$. The 90% and 2% targets respectively give estimations of 4.6% and 4.2%. This provides a simple way to estimate the hemi-reflectance of the baffle, making the assumption that its reflectance is Lambertian as for the Spectralon sample.

### Supplementary Information

Supplementary Video 1.

## Data Availability

The data that support the findings of this study are available from the corresponding author upon reasonable request.

## References

[1] Fest E (2013). Stray Light Analysis and Control.

[2] Breault RP, Bass M, Van Stryland WW, Williams DR, Wolfe WL (1995). Control of stray light, Chap 38. Handbook of Optics.

[3] Baglin, A. *et al.* CoRoT: Description of the mission and early results. In *Proceedings of the “ESO Astrophysics Symposia”* (eds Fridlund, M. *et al.*) 71 (2006).

[4] Auvergne M, Bodin P, Boisnard L (2009). The CoRoT satellite in flight: Description and performance. Astron. Astrophys..

[5] European Space Agency. *COROT Instruments*. ESA. https://www.esa.int/Science_Exploration/Space_Science/COROT/COROT_instruments. Accessed 11 Jan 2023.

[6] Plesseria, J.-Y. *et al.* Optical and mechanical design of a straylight rejection baffle for CoRoT. In *Proceedings of the SPIE 5170, Techniques and Instrumentation for Detection of Exoplanets* (2003). 10.1117/12.506726.

[7] Plesseria, J.-Y. *et al.* Straylight analysis of the external baffle of COROT. In *Proceedings of the SPIE 10568, International Conference on Space Optics—ICSO 2004*), 105680Y (2017). 10.1117/12.2307976.

[8] Rando, N. *et al.* CHEOPS, the ESA mission for exo-planets characterization: Early operations and commissioning results. In *Proceedings of the SPIE 11443, Space Telescopes and Instrumentation 2020: Optical, Infrared, and Millimeter Wave*, 1144314 (2020). 10.1117/12.2567296.

[9] Clermont L, Michel C, Stockman Y (2022). stray light correction algorithm for high performance optical instruments: The case of Metop-3MI. Remote Sens..

[10] Clermont, L. *et al.* Stray-light calibration and correction for the MetOp-SG 3MI mission. In *Proceedings of the SPIE*, vol. 10704, 1070406 (2018).

[11] Abdon, S. *et al.* Digital correction of residual straylight in FLEX images. In *Proceedings of the SPIE*, vol. 11180, 111804G (2019).

[12] Montrone, L. *et al.* Technological innovation for the ALTIUS atmospheric limb sounding mission. In *Proceedings of the SPIE*, vol. 11151, 111510S (2019).

[13] Mora, A., Biermann, M., Bombrun, A., Boyadjian, J., Chassat, F., Corberand, P., Davidson, M., Doyle, D., Escolar, D., Gielesen, W., Guilpain, T., Hernandez, J., Kirschner, V., Klioner, S. A., Koeck, C., Laine, B., Lindegren, L., Serpell, E., Tatry, P. & Thoral, P. Gaia: Focus, straylight and basic angle. In: *Proceedings of SPIE 9904, Space Telescopes and Instrumentation 2016: Optical, Infrared, and Millimeter Wave*, 99042D (2016). 10.1117/12.2230763.

[14] Montanaro M (2015). Toward an operational stray light correction for the Landsat 8 Thermal Infrared Sensor. Appl. Opt..

[15] Montanaro, M. *et al.* Performance of the proposed stray light correction algorithm for the thermal infrared sensor (TIRS) onboard Landsat 8. In *Proceedings of the SPIE*, vol. 9972, 99720F (2016).

[16] Venancio, L. M. G., Pachot, C., Carminati, L., Alvarez, J. L., Amiaux, J., Prieto, E., Bonino, L., Salvignol, J.-C., Short, A., Boenke, T., Strada, P. & Laureijs, R. Euclid end-to-end straylight performance assessment. In *Proceedings of the SPIE 9904, Space Telescopes and Instrumentation 2016: Optical, Infrared, and Millimeter Wave*, 99040P (2016). 10.1117/12.2232916.

[17] Borlaff, A., Gómez-Alvarez, P., Altieri, B., Marcum, P., Vavrek, R. *et al.* Euclid preparation. In *XVI.Exploring the ultra-low surface brightness Universe with Euclid/VIS. Astronomy and Astrophysics—A&A, EDP Sciences*, vol. 657, A92 (2022). 10.1051/0004-6361/202141935.

[18] European Space Agency. *Seeking Euclid's Hidden Stars: Commissioning Looks Up*. https://www.esa.int/Science_Exploration/Space_Science/Euclid/Seeking_Euclid_s_hidden_stars_commissioning_looks_up. Retrieved 29 Sept 2023. Accessed: 01 Nov 2023.

[19] Mazy, E., Stockman, Y. & Hellin, M. L. Design and modelisation of a straylight facility for space optical instrument. In *Proceedings of the SPIE 8550, Optical Systems Design 2012*, 855007 (2012). 10.1117/12.981159.

[20] Stockman, Y., Hellin, M. L., Marcotte, S., Mazy, E., Versluys, J., François, M., Taccola, M. & Zuccaro Marchi, A. Conceptual design of a stray light facility for Earth observation satellites. In *Proceedings of the SPIE 10564, International Conference on Space Optics—ICSO 2012*, 1056402 (2017). 10.1117/12.2309193.

[21] Clermont L, Uhring W, Georges M (2021). Stray light characterization with ultrafast time-of-flight imaging. Sci. Rep..

[22] Bradley D, Liddy B, Sleat E (1971). Direct linear measurement of ultrashort light pulses with a picosecond streak camera. Opt. Commun..

[23] Clermont, L., Uhring, W., Georges, M., Khaddour, W., Blain, P. & Mazy, E. A new paradigm in the field of stray light control and characterization enabled by ultrafast time-of-flight imaging. In *Proceedings of the SPIE 12188, Advances in Optical and Mechanical Technologies for Telescopes and Instrumentation V*, 121881F (2022). 10.1117/12.2629300.

[24] Khaddour W, Uhring W, Dadouche F, Dumas N, Madec M (2023). Calibration methods for time-to-digital converters. Sensors.

[25] Gariepy G (2015). Single-photon sensitive light-in-fight imaging. Nat. Commun..

[26] Morimoto K (2020). Megapixel time-gated SPAD image sensor for 2D and 3D imaging applications. Optica.

[27] Ummel, A., Roulet, J.-C., Holzer, J., Droz, F., Weigel, T., Clermont, L., Georges, M., Abrantes, J., Tomuta, D., Isaak, K., Doyle, D., Miranda, M. & Pache, C. Verification of straylight rejection of optical science payloads using a pulsed laser source. In *Proceedings of the SPIE 12777, International Conference on Space Optics—ICSO 2022*, 127774O (2023). 10.1117/12.2690880.

[28] Coddington I, Swann WC, Nenadovic L, Newbury NR (2009). Rapid and precise absolute distance measurements at long range. Nat. Photon..

[29] Amann M-C, Bosch TM, Lescure M, Myllylae RA, Rioux M (2001). Laser ranging: A critical review of unusual techniques for distance measurement. Opt. Eng..

[30] Abramson N (1978). Light-in-flight recording by holography. Opt. Lett..

[31] Abramson N (1983). Light-in-flight recording: High-speed holographic motion pictures of ultrafast phenomena. Appl. Opt..

[32] Kubota T, Komai K, Yamagiwa M, Awatsuji Y (2007). Moving picture recording and observation of three-dimensional image of femtosecond light pulse propagation. Opt. Express.

[33] Kakue T (2011). Moving picture recording and observation of visible femtosecond light pulse propagation. Jpn. J. Appl. Phys..

[34] Inoue T, Nagao K, Nishio K, Kubota T, Awatsuji Y (2022). Ultrafast double motion-picture recording technique for propagating light pulses with an ultrashort time difference. Opt. Lett..

[35] Inoue T, Aoyama T, Sawashima Y, Nishio K, Kubota T, Awatsuji Y (2022). Motion picture of magnified light pulse propagation with extending recordable time of digital light-in-flight holography. Appl. Opt..

[36] Inoue T, Kakue T, Nishio K, Kubota T, Awatsuji Y (2023). Multiple motion picture recording in light-in-flight recording by holography with an angular multiplexing technique. J. Opt. Soc. Am. A.

[37] Takakura, H., *et al.* Straylight identification of a crossed-Dragone telescope by time-gated near-field antenna pattern measurements. In *Proceeding of SPIE, SPIE Astronomical Telescopes, Montreal* (2022).

[38] Matsumura T, Akiba Y, Borrill J (2014). Mission design of LiteBIRD. J. Low Temp. Phys..

[39] Khan I, Lequime M, Zerrad M, Amra C (2021). Detection of ultralow light power back-reflected or back-scattered by optical components using balanced low-coherence interferometry. Phys. Rev. Appl..

[40] Clermont L, Michel C, Blain P, Loicq J, Stockman Y (2020). Stray light entrance pupil: An efficient tool for stray light characterization. Opt. Eng..

